# Supplementary material for: Causal Relationship between Aging and Anorexia Nervosa: A White-Matter-Microstructure-Mediated Mendelian Randomization Analysis
Source: Biomedicines. 2024 Aug 16;12(8):1874. doi: 10.3390/biomedicines12081874 (PMC11351342; doi:10.3390/biomedicines12081874)
Supplement: Supplementary file 1 [file biomedicines-12-01874-s001.zip › Supplementary Figure/Figure S4/Singe SNP plot of MR analysis for TL to ubm-b-1682.pdf]

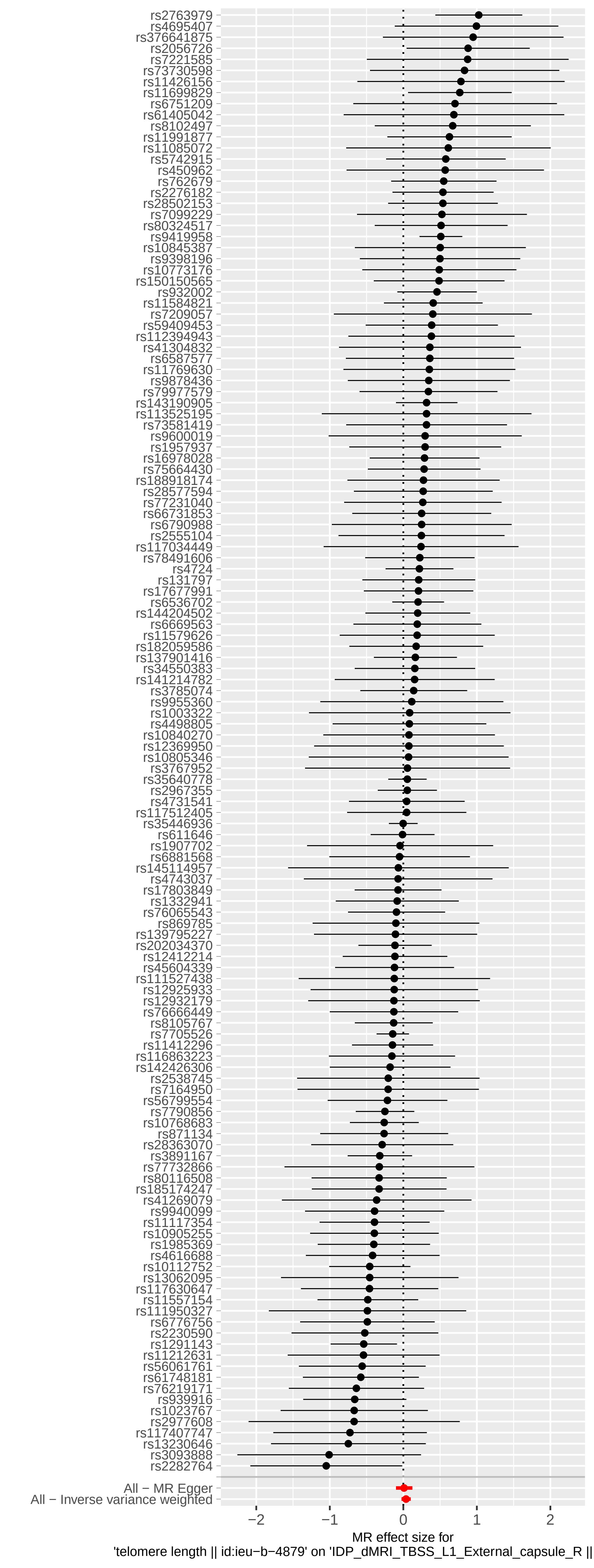

rs2763979
rs4695407
rs376641875
rs2056726
rs7221585
rs73730598
rs11426156
rs11699829
rs6751209
rs61405042
rs8102497
rs11991877
rs11085072
rs5742915
rs450962
rs762679
rs2276182
rs28502153
rs7099229
rs80324517
rs9419958
rs10845387
rs9398196
rs10773176
rs150150565
rs932002
rs11584821
rs7209057
rs59409453
rs112394943
rs41304832
rs6587577
rs11769630
rs9878436
rs79977579
rs143190905
rs113525195
rs73581419
rs9600019
rs1957937
rs16978028
rs75664430
rs188918174
rs28577594
rs77231040
rs66731853
rs6790988
rs2555104
rs117034449
rs78491606
rs4724
rs131797
rs17677991
rs6536702
rs144204502
rs6669563
rs11579626
rs182059586
rs137901416
rs34550383
rs141214782
rs3785074
rs9955360
rs1003322
rs4498805
rs10840270
rs12369950
rs10805346
rs3767952
rs35640778
rs2967355
rs4731541
rs117512405
rs35446936
rs611646
rs1907702
rs6881568
rs145114957
rs4743037
rs17803849
rs1332941
rs76065543
rs869785
rs139795227
rs202034370
rs12412214
rs45604339
rs111527438
rs12925933
rs12932179
rs76666449
rs8105767
rs7705526
rs11412296
rs116863223
rs142426306
rs2538745
rs7164950
rs56799554
rs7790856
rs10768683
rs871134
rs28363070
rs3891167
rs77732866
rs80116508
rs185174247
rs41269079
rs9940099
rs11117354
rs10905255
rs1985369
rs4616688
rs10112752
rs13062095
rs117630647
rs11557154
rs111950327
rs6776756
rs2230590
rs1291143
rs11212631
rs56061761
rs61748181
rs76219171
rs939916
rs1023767
rs2977608
rs117407747
rs13230646
rs3093888
rs2282764
All − MR Egger
All − Inverse variance weighted
−2
−1
0
1
2
MR effect size for
'telomere length || id:ieu−b−4879' on 'IDP_dMRI_TBSS_L1_External_capsule_R ||
